# Selenium Biofortification Impacts the Nutritive Value, Polyphenolic Content, and Bioactive Constitution of Variable Microgreens Genotypes

**DOI:** 10.3390/antiox9040272

**Published:** 2020-03-25

**Authors:** Antonio Pannico, Christophe El-Nakhel, Giulia Graziani, Marios C. Kyriacou, Maria Giordano, Georgios A. Soteriou, Armando Zarrelli, Alberto Ritieni, Stefania De Pascale, Youssef Rouphael

**Affiliations:** 1 Department of Agricultural Sciences, University of Naples Federico II, 80055 Portici, Italy; antonio.pannico@unina.it (A.P.); Nakhel_Christophe@hotmail.com (C.E.-N.); maria.giordano@unina.it (M.G.); depascal@unina.it (S.D.P.); 2 Department of Pharmacy, University of Naples Federico II, 80131 Naples, Italy; giulia.graziani@unina.it (G.G.); alberto.ritieni@unina.it (A.R.); 3 Department of Vegetable Crops, Agricultural Research Institute, 1516 Nicosia, Cyprus; m.kyriacou@ari.gov.cy (M.C.K.); soteriou@ari.gov.cy (G.A.S.); 4 Department of Chemical Sciences, University of Naples Federico II, 800126 Naples, Italy; zarrelli@unina.it

**Keywords:** coriander, green and purple basil, tatsoi, carotenoids, phenolic compounds, Orbitrap LC-MS/MS, sodium selenite, RDA, hidden hunger, dietary supplements

## Abstract

Selenium (Se) is considered essential for human nutrition as it is involved in the metabolic pathway of selenoproteins and relevant biological functions. Microgreens, defined as tender immature greens, constitute an emerging functional food characterized by overall higher levels of phytonutrients than their mature counterparts. The nutraceutical value of microgreens can be further improved through Se biofortification, delivering Se-enriched foods and potentially an enhanced content of bioactive compounds. The current study defined the effect of sodium selenate applications at three concentrations (0, 8, and 16 μM Se) on the bioactive compounds and mineral content of coriander, green basil, purple basil, and tatsoi microgreens grown in soilless cultivation. Analytical emphasis was dedicated to the identification and quantification of polyphenols by UHPLC-Q-Orbitrap-HRMS, major carotenoids by HPLC-DAD, and macro micro-minerals by ICP-OES. Twenty-seven phenolic compounds were quantified, of which the most abundant were: Chlorogenic acid and rutin in coriander, caffeic acid hexoside and kaempferol-3-*O*(caffeoyl) sophoroside-7-*O*-glucoside in tatsoi, and cichoric acid and rosmarinic acid in both green and purple basil. In coriander and tatsoi microgreens, the application of 16 μM Se increased the total phenols content by 21% and 95%, respectively; moreover, it improved the yield by 44% and 18%, respectively. At the same Se dose, the bioactive value of coriander and tatsoi was enhanced by a significant increase in rutin (33%) and kaempferol-3-*O*(feruloyl)sophoroside-7-*O*-glucoside (157%), respectively, compared to the control. In green and purple basil microgreens, the 8 μM Se application enhanced the lutein concentration by 7% and 19%, respectively. The same application rate also increased the overall macroelements content by 35% and total polyphenols concentration by 32% but only in the green cultivar. The latter actually had a tripled chicoric acid content compared to the untreated control. All microgreen genotypes exhibited an increase in the Se content in response to the biofortification treatments, thereby satisfying the recommended daily allowance for Se (RDA-Se) from 20% to 133%. The optimal Se dose that guarantees the effectiveness of Se biofortification and improves the content of bioactive compounds was 16 μM in coriander and tatsoi, and 8 μM in green and purple basil.

## 1. Introduction

Microgreens have recently increased in popularity by earning the title of “functional food” or “superfood” [1,2]. They are defined as tender immature greens produced from seedlings of vegetables, grains, herbs, and wild species, harvested at soil level upon the appearance of a pair of first true leaves, which usually occurs within 7–21 days from sowing, depending on the species [3,4]. Microgreens contain higher levels of phytonutrients and secondary metabolites, such as microelements, carotenoids, and phenolic compounds, than their mature-leaf counterparts [5,6]. Interestingly, the demand for microgreens is increasing both in response to the intrinsic characteristics of the product (unique intense flavor, delicate texture, vivid colors) and due to the influence of new gastronomic trends sponsored by star chefs [7], especially for their nutraceutical value and putative beneficial effects on human health [2,8]. The nutraceutical value of microgreens may be further enhanced through biofortification with micronutrients. Selenium (Se) applications have been shown to have the dual effect of both enriching food with an essential microelement and increasing the overall content of bioactive compounds in microgreens [9,10].

Selenium is deemed essential for mammal nutrition as it is involved in the metabolic pathway of selenoproteins, selenoenzymes, and thyroid hormones, with fundamental biological functions [11]. Human micronutrient deficiency, also known as “hidden hunger”, affects more than a billion people in the world [12], especially in areas with soils containing low levels of these trace elements or chemical-physical characteristics that limit their bioavailability [13]. Se deficiency leads to serious human disorders, such as heart diseases, viral infections, hyperthyroidism, diabetes, Keshan disease, and cancer [14,15,16]. These illnesses are most prevalent in developing countries, where the recommended dietary allowance (RDA) of Se (55 μg day^−1^) is not easily reached [17]. Additionally, Se supplementation with up to 400 μg day^−1^ has proven beneficial toward reducing the risk of certain types of cancer (i.e., colon, lung, and prostate cancer) in developed countries [18,19].

Opinions about selenium’s role in higher plants diverge between being considered as a non-essential element [16,20], or defined as beneficial [21,22]. Selenium application at low doses improves tolerance to oxidative stress, delays senescence, and stimulates yield, as demonstrated in several previous studies [23,24]. Selenium enhances the activity of antioxidant enzymes, such as lipoxygenase, catalase, ascorbate, and glutathione peroxidase, which in turn elicit the synthesis of secondary metabolites, including carotenoids, phenols, flavonoids, and vitamins [10,25,26]. Plants assimilate Se mainly in the form of selenate or selenite, the former being transported actively by sulphate transporters [27], while the latter is taken up via phosphate transporters [28]. However, compared to selenate, selenite is less soluble, more phytotoxic, and more difficult to transport and accumulate in plant tissues; for this reason, the selenate form is preferred for biofortification [29,30]. In any case, selenium at high concentrations depresses plant growth by interfering with the sulfur metabolic pathway. Indeed, Se phytotoxicity is mainly due to the incorporation of selenocysteines and selenomethionines, which replace their plant sulfur protein analogues [31].

Among the various biofortification techniques, Se application in the nutrient solution of soilless cultivation systems (SCSs) is certainly the most efficient solution [32,33]. In SCSs, eustress (i.e., positive stress) can be applied more reliably through accurate management of the composition and concentration of the nutrient solution [33,34]. By adopting such a technique, Se spread in the environment is minimized and Se assimilation is maximized by the absence of soil interactions; moreover, the constant root exposure to the nutrient solution affords the application of reduced Se rates, facilitates more effective standardization of food quality, and guarantees the safety of the food product offered to the consumer [32,33].

Although many Se biofortification studies have covered a wide range of cereal and vegetable crops, including wheat [35], rice [36], tomato [37], potato [38], beans [39], pea [40], endive [41], broccoli [42], rocket [27], lettuce [43], garlic [44], and shallot [45], the biofortification of microgreens with selenium has been poorly studied, aside from a few works on buckwheat [46], wheat [10], and basil [9]. Nevertheless, the effect of Se biofortification on the phenolic profile of microgreens has never been investigated. On the other hand, in a recent work where the phytochemical composition of 13 microgreens genotypes belonging to 5 botanical families was assessed, it was highlighted that coriander, basil, and tatsoi had the highest content of phenolic compounds [2].

Taking these findings into consideration, the effect of Se applications at three different concentrations was evaluated in the present study on coriander, green basil, purple basil, and tatsoi microgreens grown in SCS. The aim of the work was to illustrate Se accumulation in microgreens and identify the Se dose that enhances the nutraceutical characteristics of the product in terms of the mineral content, carotenoids, and phenolic compounds, without deleterious effects on yield.

## 2. Materials and Methods

### 2.1. Plant Material and Growth Chamber Conditions

Four microgreens genotypes were chosen to assess their bioactive compounds and mineral content: Coriander (*Coriandrum sativum* L.; Micro Splits, CN Seeds Ltd., Pymoor, Ely, Cambrigeshire, UK), two different basil cultivars (green basil, *Ocimum basilicum* L.; Orto del Sole, Bari, Puglia, Italy and purple basil, *Ocimum basilicum* L.; Condor Seed Production, Yuma, AZ, USA), and tatsoi (*Brassica rapa* L. subsp. *narinosa*; Condor Seed Production, Yuma, AZ, USA).

The experiment was carried out at the Department of Agricultural Sciences of University of Naples Federico II, Portici, Italy in a growth chamber (KBP-6395F, Termaks, Bergen, Norway) equipped with an Light-Emitting Diode (LED) panel unit (K5 Series XL750, Kind LED, Santa Rosa, CA, USA) with an emission wavelength range of 400–700 nm. The microgreens were sown on a capillary mat (Aquamat capillary matting, Premier Netting, Norfolk, UK) laid in plastic trays (14 × 19 × 6 cm: W × L × D). The sowing density was 4 seeds cm^−2^ for coriander and 7 seeds cm^−2^ for tatsoi, green, and purple basil; harvest was performed 12 days after sowing (DAS) for tatsoi and 19 DAS for coriander, green, and purple basil.

The LED panel positioned inside the chamber ensured a homogeneous distribution of light (300 ± 10 μmol m^−2^ s^−1^ at the canopy level) over the entire shelf surface (0.4 m^2^). Nevertheless, the trays were randomly rotated every 24 h in order to improve the uniformity of light intensity at the canopy level. The photosynthetic photon flux density (PPFD) and the light spectra were regulated at the canopy level using a spectral radiometer (MSC15, Gigahertz-Optik, Turkenfeld, Germany). A day/night temperature and relative air humidity of 24/18 °C and 75/85%, respectively, were established with a 16/8 h photoperiod.

Microgreens were fertigated with a modified quarter-strength Hoagland nutrient solution prepared with distilled water containing: 2.0 mM nitrate, 0.25 mM sulfur, 0.20 mM phosphorus, 0.62 mM potassium, 0.75 mM calcium, 0.17 mM magnesium, 0.25 mM ammonium, 20 μM iron, 9 μM manganese, 0.3 μM copper, 1.6 μM zinc, 20 μM boron, and 0.3 μM molybdenum, with an electrical conductivity (EC) of 0.35 dS m^−1^ and a pH of 6.0. The nutrient solution was supplied on a daily basis onto the substrate using a laboratory beaker.

The test was conducted according to a randomized design in a factorial arrangement (3 × 4), with three concentrations (0, 8, or 16 μM) of sodium selenate (Sigma-Aldrich, St. Louis, MO, USA) in the nutrient solution and four microgreen genotypes (coriander, green basil, purple basil, and tatsoi), with three replicates.

### 2.2. Plant Material and Growth Chamber Conditions

All microgreens were collected at the appearance of the second true leaf by cutting the seedlings at the substrate level with sterilized scissors. The collected material was weighed to determine the fresh yield, expressed as kg fresh weight (fw) per square meter. Fresh samples of microgreens randomly selected from each tray were instantly frozen in liquid nitrogen and stored at −80 °C before lyophilization in a freeze drier (Christ, Alpha 1-4, Osterode, Germany), in order to be used for mineral and phytochemical analyses.

### 2.3. Dry Matter and Nitrate Content Analysis

Dry weight (dw) was measured on an analytical balance (Denver instruments, Denver, CO, USA) following lyophilization until a constant weight was reached. Dry matter content was determined in triplicate based on the official method 934.01 of the Association of Official Analytical Chemists, and expressed as a percentage. The dry samples were finely ground in a Wiley Mill (MF 10.1, IKA, Staufen, Germany) to be utilized for chemical analysis. Nitrate content was analyzed by ion chromatography (ICS-3000, Dionex, Sunnyvale, CA, USA) as previously described by Kyriacou et al. [2]. Separation was achieved in gradient mode on an AS11-HC analytical column (4 × 250 mm, Dionex, Sunnyvale, CA, USA) equipped with an AG11-HC pre-column (4 × 50 mm, Dionex, Sunnyvale, CA, USA) and a dynamically regenerated suppressor DRS600 (Dionex, Sunnyvale, CA, USA). Results were expressed as mg kg^−1^ fresh weight according to the dry matter content of each sample.

### 2.4. Mineral Analysis by ICP-OES and Consumer Safety of Se-Enriched Microgreens

Macro (P, K, Ca, Mg, and Na) and trace (Fe, Zn, Mn, and Se) elements in microgreen samples were measured using an inductively coupled plasma mass spectrometer (ICP-OES Spectroblue, Spectro Ametek, Berwyn, PA, USA). A full description of the method used for the heavy metal analyses in leaf samples appears in Volpe et al. [47]. Briefly, 1000 mg of lyophilized microgreens were fully digested in a microwave digestion system (MLS-1200 Microwave Laboratory Systems, Milestone, Shelton, CT, USA) with the addition of a mixture of HNO_3_ (65%) and HCl (37%) (9:3, *v/v*; 12 mL), and the resulting solutions were transferred to 100-mL volumetric flasks and diluted to the fixed volume (50 mL) with ultrapure water (Milli-Q, Merck Millipore, Darmstadt, Germany). The calibration curve was prepared using a working standard solution with concentrations ranging from 1.0 to 100 μg L^−1^ for all non-alkaline elements (Fe, Zn, Mn, and Se) and with concentrations ranging from 100 μg L^−1^ to 10 mg L^−1^ for all alkaline elements (K, Ca, Mg, and Na). The results were expressed as mg g^−1^ dw (P, K, Ca, Mg, and Na) and μg g^−1^ dw (Fe, Zn, Mn, and Se).

The green vegetables’ hazard quotient (HQ_gv_) of selenium was calculated according to the United States Environmental Protection Agency (USEPA) Protocol [48] using the following formula:

HQ_gv_ = (ADD/RfD),

where ADD is the average daily dose of selenium (μg Se day^−1^) and RfD represents the recommended dietary tolerable upper intake level of selenium (μg Se day^−1^) assessed as equal to 400 μg day^−1^ [17], referring to the risk to human health of a 70-kg adult resulting from Se intake through the consumption of a 10-g portion of fresh microgreens. HQgv values below 1.00 indicate that the vegetable is safe for consumption by human beings.

### 2.5. Separation and Quantification of Carotenoids by HPLC-DAD

Lutein and β-carotene were extracted from 100 mg of lyophilized sample suspended in 6 mL of ethanol containing 0.1% Butylated hydroxytoluene (BHT), according to the procedure described by Kyriacou et al. [2]. Carotenoids’ separation was achieved in gradient mode using a Shimadzu HPLC (Model LC 10, Shimadzu, Osaka, Japan) equipped with a reverse phase column (250 × 4.6 mm, 5 μm Gemini C18, Phenomenex, Torrance, CA, USA). The injection volume was 20 μL while the total runtime was 25 min. The quantification was carried out by measuring the absorbance at 450 nm, against linear calibration curves built with lutein and β-carotene external standards (ranging from 5 to 100 μg mL^−1^) containing not less than six concentration levels. The results were expressed as mg kg^−1^ dw.

### 2.6. Extraction and Analysis of Polyphenols by UHPLC-Q-Orbitrap HRMS

Polyphenols were extracted from 100 mg of lyophilized microgreens following the methods detailed in Kyriacou et al. [2]. Polyphenols were quantified and separated using an UHPLC system (Thermo Fisher Scientific, Waltham, MA, USA) equipped with a quaternary pump (Ultimate 3000, Dionex, Sunnyvale, CA, USA) and a thermostated column (100 × 2.1 mm, Kinetex 1.7 µm biphenyl, Phenomenex, Torrance, CA, USA). Mass spectrometry analysis was facilitated by a Q Exactive Orbitrap LC-MS/MS (Thermo Fisher Scientific, Waltham, MA, USA). The acquisition of polyphenolic compounds was performed according to Kyriacou et al. [2], where the protocol is fully detailed. The calibration and accuracy of the equipment was monitored by using a reference standard mixture (Thermo Fisher Scientific, Waltham, MA, USA). Data analysis and processing were developed with the Xcalibur software, version 3.0.63 (Thermo Fisher Scientific, Waltham, MA, USA).

### 2.7. Statistics of Experimental Data

Data were initially subjected to a two-way analysis of variance (ANOVA). Interactions were further addressed through genotype-specific one-way ANOVA and treatment means were compared using Duncan’s multiple range test performed at *p* ≤ 0.05 using the SPSS 20 software package (IBM, Armonk, NY, USA).

## 3. Results and Discussion

The initial two-way ANOVA of genotype vs. Se treatment revealed widespread interaction for almost all variables examined (Table 1), reflecting the differential capacity of the various microgreen genotypes for Se uptake. Therefore, further one-way ANOVA and mean comparisons were performed within each genotype with respect to the Se treatments.

### 3.1. Fresh Biomass Yield and Dry Matter Content

**Figure 1:**
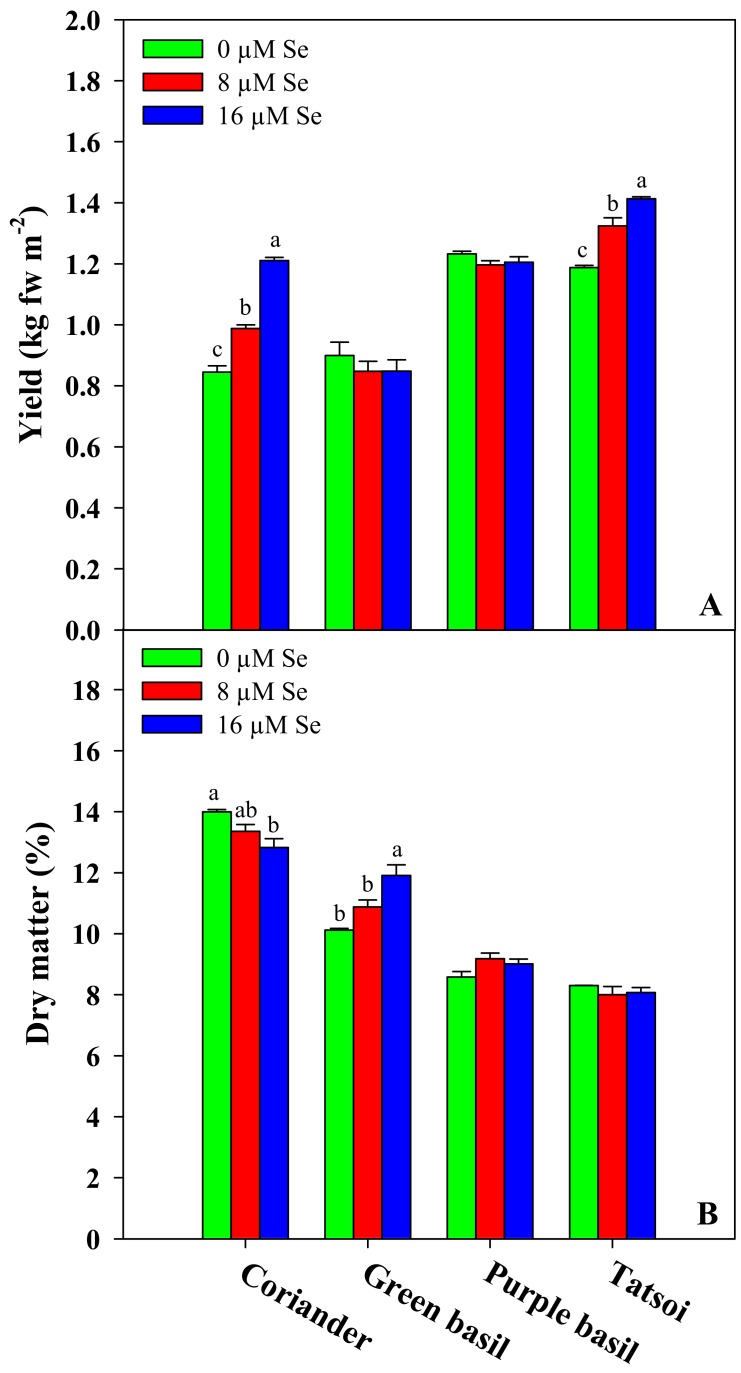
Effects of the selenium concentration on the fresh yield (**A**) and dry matter (**B**) of four microgreen genotypes grown in a growth chamber on a capillary mat substrate under three Se concentrations applied in the nutrient solution. The different letters (a–c) above the bars indicate significant mean differences within each genotype according to Duncan’s multiple range tests (*p* ≤ 0.05). The absence of letters denotes the absence of significant differences. The values are the means of three replicates. Vertical bars indicate ± SE of means.

Tatsoi was the earliest genotype achieving harvest maturity (12 DAS), while coriander, green, and purple basil were harvested exactly a week later (19 DAS). The growth period of the respective microgreens is comparable with that found in a previous work on the same genotype harvested at the second true leaf stage, in which tatsoi was found to be harvest ready earlier than coriander, green, and purple basil [2]. Yield varied significantly between genotypes (Figure 1), with the highest fresh biomass achieved by tatsoi (1.19–1.41 kg m^−2^), followed by purple basil (1.20–1.23 kg m^−2^), coriander (0.84–1.21 kg m^−2^), and green basil (0.85–0.90 kg m^−2^). On the other hand, the dry matter content was highest in coriander (12.8–14.0%), followed by green basil (10.1–11.9%), purple basil (8.6–9.2%), and tatsoi (8.0–8.3%; Figure 1B). Regardless of the genotype, yield was overall lower than previously found by Kyriacou et al. [2] on the same microgreen genotypes sown at an equal seed density on peat-based substrate. This response is attributable to the different growth substrates used; the commercial peat-based media provide an optimal aeration-moisture root environment and a more efficient supply of nutrients, promoting faster growth than the chemically inert capillary mat presently used. In our study, no toxic effect of Se addition on the fresh yield of microgreens occurred; by contrast, the dry matter content observed in our experiment was overall higher than that reported by Kyriacou et al. [2], owing to the slower growth sustained on the capillary mat.

Selenium treatments significantly improved the yield of tatsoi and coriander. In particular, the addition of Se at 8 and 16 μM increased the fresh biomass of tatsoi by 11% and 18%, respectively, and increased the coriander fresh biomass by 18% and 44%, respectively (Figure 1A). No significant effect of Se treatment was observed on the fresh yield of green and purple basil (Figure 1A). Nevertheless, in green basil, the dry matter content increased by 8% and 18% at the 8 and 16 μM Se rates, respectively, while an opposite effect was observed in coriander at the 16 μM Se treatment with a reduction of 8% compared to the control. However, no significant Se effect on dry matter was found in purple basil and tatsoi. No deleterious effect of Se treatments was observed on the fresh yield of the four microgreen genotypes studied. The non-significant effect on yield observed in green and purple basil was in agreement with previous studies on Se-biofortified microgreens [9] and mature plants [26] of basil. On the other hand, the beneficial Se effect found on the yield of coriander and tatsoi was consistent with the growth-promoting response to low Se doses demonstrated in previous works on rice sprouts [49] and mature plants of Indian mustard [50], ryegrass [51], spinach [52], and lettuce [53]. It was demonstrated that Se at low concentrations can stimulate plant growth, enhance photosynthesis, and support the homeostasis of essential nutrients [54]. In addition, low-Se-dose treatments work as a stress modulator that inhibits the accumulation of Reactive Oxygen Species (ROS) during stress by acting as an antioxidant [55]. On the contrary, at high concentrations, selenium acts as a pro-oxidant, inducing an increase in ROS and lipid peroxidation [56,57]. Considering that the Se response is dose as well as genotype dependent, further studies should focus on clarifying the interaction between genotype and application dose, in order to identify the combination that guarantees an optimal balance between yield and biofortification in targeted species/genotypes.

### 3.2. Nitrate Content and Mineral Composition

One of the most critical factors related to the consumption of fresh leafy vegetables is certainly associated with their nitrate content. Indeed, high nitrate levels can be harmful to human health, causing serious physiological disorders, such as methemoglobinemia and blue baby syndrome [58]. The maximum limit for the nitrate concentration established by the Commission Regulation (EU) No 1258/2011 [59] for the commercialization of fresh mature vegetables ranges from 3000 to 7000 mg kg^−1^ fw depending on the species (lettuce, spinach, and rocket) and the harvest season (summer or winter). In the present work, the nitrate content was significantly different among microgreen genotypes; in particular, purple basil was the highest nitrate accumulator (451.2–539.9 mg kg^−1^ fw) followed by green basil (84.1–216.1 mg kg^−1^ fw), tatsoi (63.7–80.6 mg kg^−1^ fw), and coriander (9.5–18.5 mg kg^−1^ fw) (Table 2). In any case, although microgreens have not yet been regulated regarding nitrate content, the levels found were far below the limits established by the EU regulation for mature lettuce, spinach, and rocket salad crops. The microgreen nitrate content observed in our experiment was overall much lower than the values (2549–4032 mg kg^−1^ fw) previously reported by Kyriacou et al. [2]. This remarkable difference could be related to the higher content and release of nitrate from the peat-based substrate used in the work of Kyriacou et al. [2] compared to the chemically inert capillary mat adopted in the current experiment. Regardless of the genotypes, selenium applications have significantly affected the nitrate content of microgreens (Table 2). Overall, Se applications at both doses demonstrated an average 24% reduction of nitrate levels in all four genotypes compared to the untreated control. This important result could be associated with the antagonistic effect of selenate exerted on nitrate anions [60]. In addition, Nowak and co-authors [61] demonstrated that Se enhanced nitrate reductase activity in plants, leading to a decrease of the foliar nitrate content. Our data are consistent with the nitrate reduction observed in previous works on lettuce plants treated with selenate at different concentrations [43,62,63].

Major (P and K), secondary (Ca and Mg), and trace (Na, Fe, Zn, and Mn) minerals are essential for human health as components of our skeletal structure and are essential for countless body processes, acting as co-factors for several enzymes [64]. Among the four microgreen genotypes examined, significant differences were observed relative to the concentrations of macro (P, K, Ca, Mg, and Na) and micro (Fe, Zn, and Mn) nutrients (Table 2). Among all genotypes, the most abundant macroelement was K, ranging from 2.9 to 13.0 mg g^−1^ dw, followed by Ca (3.2–10.6 mg g^−1^ dw), P (1.9–4.5 mg g^−1^ dw), Mg (2.1–3.6 mg g^−1^ dw), and Na (0.4–0.8 mg g^−1^ dw), whereas the most representative microelements were Fe (20.5–57.2 μg g^−1^ dw), Zn (23.2–64.6 μg g^−1^ dw), and Mn (6.6–46.1 μg g^−1^ dw) (Table 2). In general, the macro and microelement profiles of microgreens tested is fully comparable to the trend described in previous works on microgreens; however, the overall mineral content is slightly lower than the microgreens grown on peat-based substrate [2,65].

In green basil, an increase of 63% and 95% of phosphorus, 39% and 40% of potassium, 22% and 30% of calcium, and 41% and 66% of magnesium was observed at doses of 8 and 16 μM Se, respectively, in comparison to the control. Likewise, a significant rise of 18% and 25% of phosphorus, 17% and 14% of potassium, 22% and 29% of calcium, and 15% and 17% of magnesium was found in purple basil at doses of 8 and 16 μM Se, respectively. In coriander, Se applications improved the concentration of P (15% and 22% at the 8 and 16 μM Se doses, respectively), K (28% at the 16 μM Se dose), Ca (32% at the 16 μM Se dose), and Mg (19% at the 16 μM Se dose) compared to the control treatment (Table 2). In contrast, an opposite effect was found in tatsoi, whereby the content of phosphorus and magnesium decreased at doses of 8 μM (23% and 36%, respectively) and 16 μM Se (14% and 23%, respectively), while the K and Ca concentration significantly decreased only at the dose of 8 μM Se by 30% and 36%, respectively, compared to the control. Regarding Na accumulation, in coriander and green basil, an increase of 18% and 14% was observed, respectively, at a dose of 16 μM Se, while on the contrary, in purple basil and tatsoi, an average reduction of 30% and 36% was noted, respectively, at both Se applications (Table 2).

Concerning the microelement contents, a significant increase of 7% and 17% of Fe, 8% and 15% of Zn, and 22% and 31% of Mn was observed in purple basil at doses of 8 and 16 μM Se, respectively, compared to the control. Likewise, a significant rise of 9% and 24% of Fe and 17% and 59% of Zn was found in coriander at doses of 8 and 16 μM Se, respectively; whereas Mn increased by 41% merely at the dose of 16 μM Se. Contrarily, in tatsoi, a decrease of 43% and 22% of Fe, 20% and 10% of Zn, and 37% and 24% of Mn was noted at doses of 8 and 16 μM Se, respectively, in comparison to the control. In green basil, the addition of selenium to the nutrient solution at both doses reduced the zinc content on average by 59% and increased the manganese concentration on average by 67%, while Fe decreased by 4% only at the dose of 8 μM Se.

To the best of our knowledge, there are currently no works in the literature regarding the effect of Se biofortification on microgreens’ mineral contents. However, the influence of Se applications on the macro and micronutrient content is not understood at all but appears to differ in a genotype-specific way. In coriander and basil (both cultivars), all macroelements tended to increase with Se treatments while an opposite effect was found in tatsoi. Our results are consistent with the findings observed in various studies on mature plants treated with selenium, in which K, P, Ca, and Mg increased or decreased depending on the species and/or cultivar [43,66,67]. Likewise, iron and manganese microelements increased in coriander and purple basil with Se applications as found by Rios et al. [67] on lettuce, while iron, zinc, and manganese significantly decreased in tatsoi according to the results found by Wu and Huang [66] on white clover.

### 3.3. Selenium Biofortification and Consumer Safety

**Figure 2:**
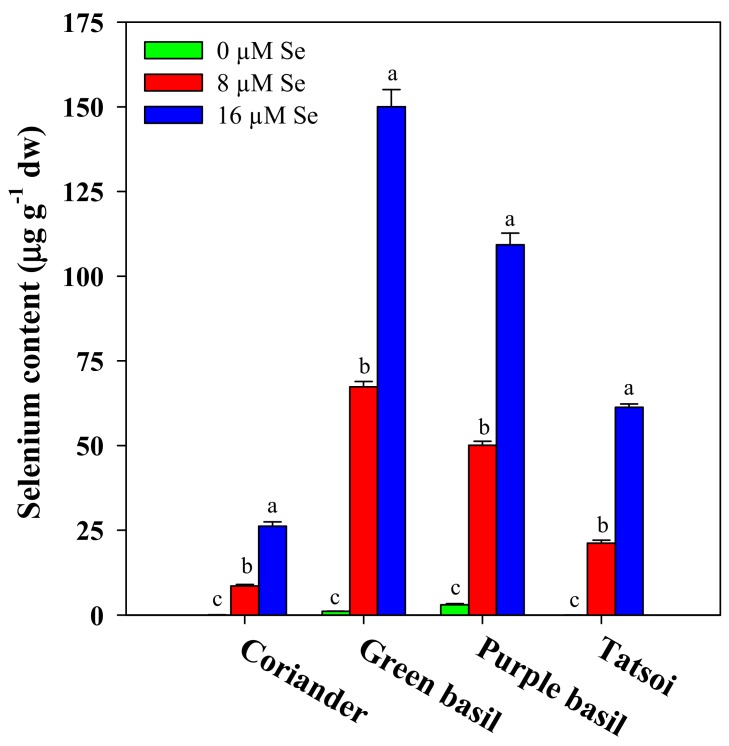
Effects of the selenium concentration on selenium biofortification of four microgreen genotypes grown in a growth chamber on a capillary mat substrate under three Se concentrations applied in the nutrient solution. The different letters (a–c) above bars indicate significant mean differences within each genotype according to Duncan’s multiple range tests (*p* ≤ 0.05). The values are the means of three replicates. Vertical bars indicate ± SE of means.

In selenium biofortification programs, it is fundamental that the selected crops are able to assimilate and accumulate this element in the edible portions of the plant. In the current study, all microgreen genotypes incurred an increase in Se content in response to the increasing Se doses applied. Across Se treatments, green basil reached the highest selenium content (72.83 μg g^−1^ dw) on average followed by purple basil (54.14 μg g^−1^ dw), tatsoi (27.54 μg g^−1^ dw), and coriander (11.60 μg g^−1^ dw) (Figure 2). In particular, selenium content gradually increased with the amount of supplied Se in all four microgreen genotypes: In coriander 0.05, 8.57, and 26.18 μg g^−1^ dw; in green basil 1.10, 67.35, and 150.03 μg g^−1^ dw; in purple basil 3.04, 50.10, and 109.28 μg g^−1^ dw; whereas in tatsoi, 0.04, 21.24, and 61.34 μg g^−1^ dw at doses of 0, 8, and 16 μM, respectively (Figure 2). Our findings were in agreement with previous works on microgreens of buckwheat [46], basil [9], and wheat [10], demonstrating the efficacy of achieving Se biofortification of microgreens. Furthermore, our findings corroborate previous findings on plant roots’ capability for selenium uptake through passive diffusion and sulfate transporters due to the chemical similarity of this element with sulfur, and then it is transferred to the shoots via xylem [16,27,28]. It is well established that selenate assimilation in plants occurs through an active transport process led by sulfate transporters (SULTRs) [27]. SULTRs mediate the sulfate movement in the vascular bundles, therefore both sulphate and selenate are actively accumulated against their electrochemical gradient in the plant cells [27,57].

Selenium has been recognized as a trace mineral essential for human health as its deficiency causes serious diseases, such as cardiovascular diseases, cancer, viral infections, and diabetes [14,15,16]. However, it is characterized by a narrow range between the deficiency level (55 μg day^−1^) and the toxicity level (400 μg day^−1^) [17,19]. The Mediterranean diet is based on cereals, vegetables, fruit, meat, and dairy products and these foods are normally sufficient to guarantee, on average, a total Se intake of about 80 μg day^−1^ per person [68]. However, the dietary habits of the Mediterranean populations are changing, thereby introducing the risk of deficiencies in particular vitamins and minerals. On the other hand, in some countries, such as Brazil, a per capita daily intake of only 25 μg Se was found among the population, requiring an integration of at least 30 μg Se day^−1^ [69]. The literature provides no data regarding the average amount of daily consumed microgreens; however, considering that microgreens are normally adopted in minute quantities in order to garnish and enhance the taste of salad mix, meat, or fish [7], the average daily serving could be assumed to be around 10 g fw. In the current study, Se daily intake increased linearly in all microgreen genotypes with the Se dose, ranging from 0.07 to 33.6 μg day^−1^ in coriander, 1.12 to 178 μg day^−1^ in green basil, 2.60 to 98.4 μg day^−1^ in purple basil, and from 0.04 to 49.5 μg day^−1^ in tatsoi (Table 3). Therefore, the percentage of RDA-Se increased with the same trend, reaching a peak at the 16 μM Se dose. Then, in countries like Brazil, the RDA can be satisfied by consuming, for example, 10 g fw day^−1^ of coriander at the 16 μM Se dose, 4 g fw day^−1^ of green basil at the 8 μM Se dose, 7 g fw day^−1^ of purple basil at the 8 μM Se dose, or 6 g fw day^−1^ of tatsoi at the 16 μM Se dose (Table 3). In addition, in order to examine the risks to human health, the green vegetables’ hazard quotient (HQgv) was calculated according to the USEPA protocol [48]. In our experiment, HQgv increased with the Se application rate, ranging overall from 0.00 to 0.45; therefore, the 10-g daily portion of biofortified microgreens can be considered safe since the values of HQgv were less than 1 in all four microgreen genotypes and for all Se doses (Table 3).

### 3.4. Target Carotenoids

Carotenoids are essential hydrophobic molecules that have a lipophilic antioxidant capacity mainly due to the conjugated double bonds of their long polyene chain, which are capable of inhibiting reactive oxygen species (ROS) and reducing oxidative damage [70]. In particular, lutein and β-carotene are the most widely distributed carotenoids in fruits and vegetables frequently consumed by the population [71]. Lutein plays an antioxidant role in macular surface membranes, as it is able to absorb any blue light striking the retina, which is thought to cause degeneration of the delicate surface membrane [72], while β-carotene, being a retinol precursor with a high conversion rate, supplies the human diet a substantial amount of vitamin A that is essential for immune function and vision [73].

**Figure 3:**
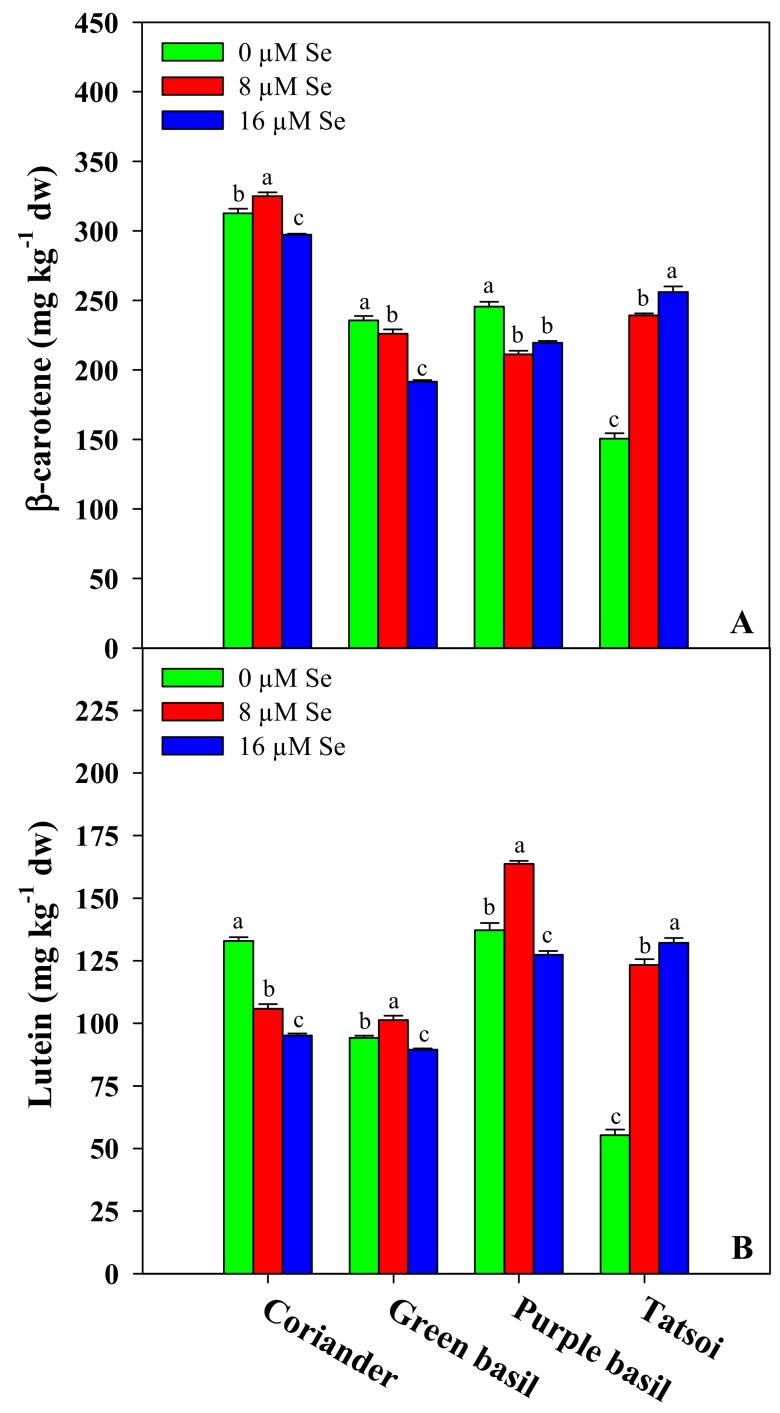
Effects of the selenium concentration on the β-carotene (**A**) and lutein (**B**) contents of four microgreen genotypes grown in a growth chamber on a capillary mat substrate under three Se concentrations applied in the nutrient solution. The different letters (a–c) above the bars indicate significant mean differences within each genotype according to Duncan’s multiple range tests (*p* ≤ 0.05). The values are the means of three replicates. Vertical bars indicate ± SE of means.

The carotenoid concentration of the microgreens was quite variable among the four genotypes; in particular, the lutein content was on average higher in purple basil (142.8 mg kg^−1^ dw) and lower in green basil (95.0 mg kg^−1^ dw), whereas β-carotene values were the highest in coriander (311.6 mg kg^−1^ dw) and the lowest in green basil (217.7 mg kg^−1^ dw) and tatsoi (215.3 mg kg^−1^ dw). In basil, Se treatment at the dose of 8 μM increased the lutein content by 7% and 19%, respectively, in the green and purple cultivars (Figure 3B). Likewise, in tatsoi, the lutein concentration was significantly higher at the dose of 8 μM Se (123%) and 16 μM Se (139%) with respect to the control, whereas in coriander, the Se treatments elicited a reduction of the same molecule by 20% and 28% at doses of 8 and 16 μM, respectively (Figure 3B). Regarding the β-carotene concentration, Se treatments reduced the content of this compound in green basil by 4% and 19% and in purple basil by 14% and 11% at doses of 8 and 16 μM, respectively (Figure 3A). On the contrary, in tatsoi, the β-carotene content was significantly higher with Se applications at doses of 8 (59%) and 16 μM (70%), while in coriander, there was an increase of 4% at the dose of 8 μM Se (Figure 3A).

To our knowledge, there is one work [40] on pea sprouts regarding the effect of Se biofortification on β-carotene and lutein contents, but there are no reports in the literature on this topic concerning microgreens. However, a significant increase in the total carotenoid content was found in Se-biofortified wheat microgreens grown in a deep flow technique hydroponic system [10]. This finding is in agreement with our results on tatsoi (both carotenoids) at both Se doses, and on coriander (β-carotene) and both basil cultivars (lutein) at the dose of 8 μM Se (Figure 3A,B). Indeed, Se applications enhance the biosynthesis of photosynthetic pigments in plants, triggering the repair of chloroplasts damaged by abiotic stress and ROS [55,74,75]. However, an opposite effect was found in rice sprouts by D’Amato et al. [49], in which both selenite and selenate applications entailed a reduction in the total carotenoid content. This latter result was consistent with the decrease of β-carotene in basil and lutein in coriander that occurred in our experiment (Figure 3A,B). On the other hand, in previous work on *Arabidopsis*, it was demonstrated that the Se supply may downregulate phytoene synthase, which is the key enzyme involved in carotenoid biosynthesis [76]. Therefore, these findings denote that the activation of molecular and physiological mechanisms influences differently the biosynthesis and accumulation of secondary metabolites according to a genotype-dependent response [31].

### 3.5. Phenolic Compounds Profiling

The Q Exactive Orbitrap LC-MS/MS analysis revealed the presence of 27 polyphenols among the four microgreen genotypes treated with Se. The chromatographic separation and quantification highlighted a substantial difference in the phenolic composition between the different microgreen genotypes. Irrespective of Se treatments, green basil had the highest level of total polyphenol detected on average (13,698 μg g^−1^ dw) closely followed by purple basil (10,830 μg g^−1^ dw) and coriander (10,237 μg g^−1^ dw), while tatsoi (594 μg g^−1^ dw) showed a much lower phenolic content (Table 4). Comparable ranges of the total polyphenols content were previously reported by Xiao et al. [8] (1500–7000 μg g^−1^ dw) and by Kyriacou et al. [2] (700–6000 μg g^−1^ dw) for peat-grown microgreens; however, the range maxima presently reported exceed those of the aforementioned works, likely owing to the slower growth rate and higher dry matter content of microgreens grown on a capillary mat. Regarding the effect of the Se treatments, in coriander and tatsoi microgreens, the application of 16 μM Se increased the total polyphenols content by 21% and 95%, respectively, compared to the untreated control. However, in green basil, the sum of detected phenolic acids was 32% higher only at the 8 μM Se application with respect to the control, while in the purple cultivar, the total polyphenols content was significantly decreased by 22% at both Se treatments.

In addition to the total quantity of polyphenols detected, the four microgreen genotypes differed specifically in the levels of key phenolic compounds. Irrespective of Se treatments, in coriander, chlorogenic acid (4516 μg g^−1^ dw) and rutin (3999 μg g^−1^ dw) were on average the most abundant; in tatsoi, the prevalent polyphenols were caffeic acid hexoside (258 μg g^−1^ dw) and kaempferol-3-*O* (caffeoyl) sophoroside-7-*O*-glucoside (126 μg g^−1^ dw); while in both green and purple basil, the most abundant on average were cichoric acid (1390 and 609 μg g^−1^ dw, respectively) and rosmarinic acid (11747 and 9950 μg g^−1^ dw, respectively) (Table 4). In coriander, a similar phenolic profile was reported in mature plants [77], with chlorogenic acid and rutin present among the major compounds. A previous work by Kyriacou et al. [2] confirmed the presence of kaempferol-3-*O* (caffeoyl) sophoroside-7-*O*-glucoside among the main phenolic compounds of tatsoi microgreens, while the caffeic acid hexoside was not detected. In the case of basil, cichoric acid and rosmarinic acid were also among the most abundant phenols present in the mature tissues of this species [78], suggesting that the phenolic transformation of basil did not incur significant changes with ontogenesis.

Significant differences in the polyphenolic profile were also observed in response to Se treatments. In coriander, the concentration of rutin, feruloyl quinic acid, luteolin-3-*O*-rutinoside, and kaempferol-3-*O*-rutinoside was higher than the untreated control at both Se doses, while quercitin-3-*O*-glucoside and dicaffeoylquinic acid increased at the lowest Se dose and ferulic acid increased only at the highest Se dose (Table 4). A similar treatment effect was found in tatsoi, in which kaempferol-3-*O*-sophoroside-7-*O*-glucoside, quercetin-sophoroside, apigenin-7-*O*-rutinoside, kaempferol-7-*O*-glucoside, and apigenin-7-rhamnoside-4-rutinoside were higher at both Se doses, while chlorogenic acid and feruloyl quinic acid increased at the 8 μM Se dose and caffeic acid hexoside, kaempferol-3-O(caffeoyl)sophoroside-7-*O*-glucoside, O(feruoyll)sophoroside-7-*O*-glucoside isorhamnetin-3-gentiobioside, kaempferol-3-O(coumaroyl)soph-7-*O*-glucoside, luteolin-7-*O*-glucoside, kaempferol-3-O(feroyll)sophoroside-7-*O*-glucoside, and coumaroyl quinic acid increased significantly only at tthee 16 μM Se dose (Table 4).

In green basil, the content of caffeic acid hexoside, luteolin-7-*O*-glucoside, feruloyl quinic acid, and cirsiliol was higher than the control at both Se doses, while cichoric acid, chlorogenic acid, caffeic acid, rosmarinic acid, ferulic acid, and apigenin-7-*O*-glucoside increased only at the lowest Se dose (Table 4). On the contrary, in purple basil, a reduction of almost all the detected phenols was found in both Se treatments with respect to the control (Table 4). The content of phenolic acids varied according to the genotype and the Se dose in a specific way. In coriander and tatsoi, our results are consistent with what was found by D’amato et al. [49], which reported a rise in phenolic compounds in rice sprouts as the applied Se dose increased. Likewise, Se treatments in the range 0.25–0.50 mg L^−1^ (3.2-6.4 μM Se) were shown to increase the total phenol content in wheat microgreen extract [10]. Regarding basil, in mature plants fertigated with a nutrient solution enriched with sodium selenate (2, 5, and 10 μM Se), Skrypnik et al. [26] found a rise in the total phenolic compound content as the Se dose increased. These findings are in agreement with our results on green basil but in contrast with what was found on purple basil microgreens. Selenium induces abiotic stress similar to that caused by other heavy metals. Se-biofortified plants react to the presence of this element by activating the phenylpropanoid pathway [79], which results in the production of phenolic compounds in order to improve ROS scavenging [80]. However, polyphenolic acids, as well as the total content of phenolic compounds, can increase or decrease depending on the Se treatment, plant growth stage, and plant species [16,49].

## 4. Conclusions

Nowadays, malnutrition still affects large portions of the world and many countries have recently resorted to biofortification programs to obtain micronutrient-enriched foods to enhance human nutrition and health. Microgreens represent an emerging class of functional foods with immense potential to improve the human diet and address nutritional deficiencies. In the present study, we proposed the Se biofortification of four microgreen genotypes to produce Se-enriched foods with a high nutraceutical profile in a very simple soilless cultivation system. Our results indicated that selenium doses up to 16 μM significantly enhanced the content of this element without depressing the yield, thus demonstrating the effectiveness of Se biofortification of microgreens. In coriander and tatsoi, the application of 16 μM Se triggered a substantial increase in the total phenols content (21% and 95%, respectively) and boosted fresh yield by 44% and 18%, respectively. Instead, in green and purple basil, Se doses of 8 μM significantly improved the lutein concentration (7% and 19%, respectively) and the overall macroelements content, as well as the total amount of polyphenols (32%) in the green pigmented cultivar. Regarding the effectiveness of Se biofortification, a fresh portion (10 g fw) of coriander and tatsoi microgreens treated with the dose of 16 μM Se satisfied 61% and 90% of the RDA-Se, respectively, whereas the 8 μM Se application was enough to supply 133% and 83% of the RDA-Se by consuming a fresh portion of basil-enriched microgreens of green and purple cultivars, respectively. In conclusion, the optimal Se dose that ensures the effectiveness of Se biofortification and additionally enhances the bioactive content of microgreens is 16 μM in coriander and tatsoi and 8 μM in green and purple basil. The potential success of vegetable biofortification is based on the right selection of target crops and on the optimization and standardization of the cultivation systems. These are the prerequisites that guarantee sustainability in the production of micronutrient-enriched, safe to eat, and high-quality food that meets the RDA standards for the targeted element, while being easy to introduce into the diet of different regions of the world.

## Figures and Tables

**Table 1:** Two-way analysis of variance (ANOVA) of all the analyzed variables of the four microgreen genotypes grown in a growth chamber on capillary mat substrate under three Se concentrations applied in the nutrient solution.

Variables	Source of Variance			Variables	Source of Variance		
	Genotype (G)	Selenium (S)	G × S		Genotype (G)	Selenium (S)	G × S
Fresh yield	***	***	***	kaempferol-3-O(caffeoyl)sophoroside-7-*O*-glucoside	***	***	***
Dry matter	***	ns	***	isorhamnetin-3-gentiobioside	na	***	na
Nitrate	***	**	ns	kaempferol-3-*O*-(coumaroyl)soph-7-*O*-glucoside	***	***	***
P	***	***	***	luteolin-7-*O*-glucoside	***	ns	***
K	***	***	***	apigenin-malonil-glucoside	***	***	***
Ca	***	***	***	kaempferol-3-O(feruoyll)sophoroside-7-*O*-glucoside	***	***	***
Mg	***	***	***	coumaroyl quinic acid	***	***	***
Na	***	***	***	rutin	***	***	***
Fe	***	***	***	apigenin-7-*O*-rutinoside	***	***	***
Zn	***	***	***	quercetin-3-*O*-glucoside	***	***	***
Mn	***	**	***	feruloyl quinic acid	***	***	***
Se	***	***	***	rosmarinic acid	***	*	***
Se intake	***	***	***	cirsiliol	***	***	***
RDA-Se	***	***	***	ferulic acid	***	***	***
HQgv	***	***	***	luteolin-3-*O*-rutinoside	***	***	***
lutein	***	***	***	kaempferol-7-*O*-glucoside	***	***	***
β-carotene	***	***	***	dicaffeoylquinic acid	***	***	***
cichoric acid	***	***	***	kaempferol-3-*O*-rutinoside	***	***	***
chlorogenic acid	***	ns	ns	quercetin-rhamnoside	***	ns	ns
caffeic acid	***	***	***	luteolin-malonil-hexose	ns	ns	ns
kaempferol-3-*O*-sophoroside-7-*O*-glucoside	na	***	na	apigenin-7-rhamnoside-4-rutinoside	na	***	na
quercetin-sophoroside	***	***	***	apigenin-7-*O*-glucoside	***	***	***
caffeic acid hexoside isomers	***	***	***				
2-xylosylvitexin	***	ns	ns	Total polyphenols	***	ns	***

ns, *, **, *** Non-significant or significant at *p* ≤ 0.05, 0.01, and 0.001, respectively. na not available.

**Table 2:** Nitrate, phosphorus (P), potassium (K), calcium (Ca), magnesium (Mg), sodium (Na), iron (Fe), zinc (Zn), and manganese (Mn) concentrations of four microgreen genotypes grown in a growth chamber on a capillary mat substrate under three Se concentrations applied in the nutrient solution.

Genotype	Selenium	Nitrate	P	K	Ca	Mg	Na	Fe	Zn	Mn
		(mg kg^−1^ fw)	(mg g^−1^ dw)	(mg g^−1^ dw)	(mg g^−1^ dw)	(mg g^−1^ dw)	(mg g^−1^ dw)	(μg g^−1^ dw)	(μg g^−1^ dw)	(μg g^−1^ dw)
Coriander	0 µM	18.5 ± 2.75 a	3.04 ± 0.08 c	7.23 ± 0.23 b	3.15 ± 0.04 b	2.17 ± 0.08 b	0.61 ± 0.01 b	20.45 ± 0.06 c	23.18 ± 0.39 c	6.61 ± 0.17 b
	8 µM	10.0 ± 0.43 b	3.49 ± 0.04 b	8.09 ± 0.39 ab	3.41 ± 0.14 b	2.36 ± 0.03 ab	0.63 ± 0.03 b	22.28 ± 0.58 b	27.22 ± 0.96 b	7.64 ± 0.59 b
	16 µM	9.5 ± 0.58 b	3.72 ± 0.03 a	9.27 ± 0.43 a	4.15 ± 0.02 a	2.58 ± 0.09 a	0.72 ± 0.01 a	25.42 ± 0.08 a	36.79 ± 0.21 a	9.30 ± 0.05 a
	Significance	*	***	*	***	*	*	***	***	**
Green basil	0 µM	216.1 ± 8.34 a	1.87 ± 0.12 c	9.19 ± 0.22 b	8.16 ± 0.49 b	2.15 ± 0.03 b	0.49 ± 0.01 b	36.73 ± 0.27 a	64.56 ± 4.52 a	13.18 ± 0.39 c
	8 µM	159.3 ± 4.05 b	3.05 ± 0.05 b	12.80 ± 0.08 a	9.95 ± 0.42 a	3.04 ± 0.07 a	0.54 ± 0.02 b	35.27 ± 0.25 b	27.88 ± 0.22 b	21.27 ± 0.33 b
	16 µM	84.1 ± 7.63 c	3.65 ± 0.21 a	12.90 ± 0.16 a	10.62 ± 0.54 a	3.56 ± 0.28 a	0.56 ± 0.01 a	36.83 ± 0.51 a	24.97 ± 1.16 b	22.85 ± 0.45 a
	Significance	***	***	***	*	**	*	*	***	***
Purple basil	0 µM	539.9 ± 13.3 a	3.48 ± 0.19 b	11.11 ± 0.47 b	7.30 ± 0.46 b	2.87 ± 0.13 b	0.67 ± 0.01 a	48.71 ± 0.63 c	35.83 ± 0.31 c	10.20 ± 0.18 c
	8 µM	451.2 ± 21.9 b	4.12 ± 0.03 a	13.03 ± 0.38 a	8.88 ± 0.34 a	3.29 ± 0.04 a	0.46 ± 0.01 b	51.93 ± 0.90 b	38.80 ± 0.16 b	12.41 ± 0.18 b
	16 µM	453.3 ± 19.8 b	4.34 ± 0.11 a	12.63 ± 0.03 a	9.44 ± 0.08 a	3.36 ± 0.07 a	0.48 ± 0.01 b	57.16 ± 0.68 a	41.36 ± 0.24 a	13.32 ± 0.25 a
	Significance	*	**	*	**	*	***	***	***	***
Tatsoi	0 µM	80.6 ± 2.98 a	4.48 ± 0.22 a	4.11 ± 0.19 a	4.42 ± 0.08 a	3.33 ± 0.20 a	0.75 ± 0.02 a	52.56 ± 3.30 a	43.22 ± 1.81 a	46.06 ± 2.40 a
	8 µM	63.7 ± 0.12 b	3.44 ± 0.06 b	2.86 ± 0.17 b	2.85 ± 0.27 b	2.12 ± 0.08 b	0.39 ± 0.02 c	29.98 ± 0.27 c	34.75 ± 0.16 c	28.81 ± 0.53 c
	16 µM	64.7 ± 0.97 b	3.84 ± 0.04 b	3.82 ± 0.15 a	3.90 ± 0.17 a	2.56 ± 0.10 b	0.58 ± 0.03 b	40.79 ± 1.61 b	38.77 ± 0.05 b	35.03 ± 1.46 b
	Significance	***	**	**	**	**	***	***	**	***

ns, *, **, *** Non-significant or significant at *p* ≤ 0.05, 0.01, and 0.001, respectively. Different letters within each column indicate significant mean differences within each genotype according to Duncan’s multiple range tests (*p* ≤ 0.05). All data are expressed as mean ± SE, *n* = 3.

**Table 3:** Se daily intake, percentage of recommended daily allowance for Se (RDA-Se), and hazard quotient (HQgv) for Se intake by adult humans (70-kg body weight), through the consumption of 10-g portions of fresh microgreens grown on a capillary mat substrate in a growth chamber under three Se concentrations applied in the nutrient solution.

Genotype	Selenium	Se Intake with 10 g fw of Microgreens	RDA-Se with 10 g fw of Microgreens	HQ_gv_ with 10 g fw of Microgreens
		(μg day^−1^)	(%)	
Coriander	0 µM	0.07 ± 0.0 c	0.12 ± 0.0 c	0.00 ± 0.00 c
	8 µM	11.5 ± 0.8 b	20.8 ± 1.5 b	0.03 ± 0.00 b
	16 µM	33.6 ± 1.6 a	61.0 ± 2.9 a	0.08 ± 0.00 a
	Significance	***	***	***
Green basil	0 µM	1.12 ± 0.1 c	2.03 ± 0.1 c	0.00 ± 0.00 c
	8 µM	73.2 ± 1.7 b	133 ± 3.1 b	0.18 ± 0.00 b
	16 µM	178 ± 5.5 a	325 ± 10 a	0.45 ± 0.01 a
	Significance	***	***	***
Purple basil	0 µM	2.60 ± 0.3 c	4.73 ± 0.6 c	0.01 ± 0.00 c
	8 µM	46.0 ± 2.0 b	83.7 ± 3.7 b	0.12 ± 0.01 b
	16 µM	98.4 ± 1.5 a	179 ± 2.6 a	0.25 ± 0.00 a
	Significance	***	***	***
Tatsoi	0 µM	0.04 ± 0.0 c	0.07 ± 0.0 c	0.00 ± 0.00 c
	8 µM	17.0 ± 1.2 b	31.0 ± 2.2 b	0.04 ± 0.00 b
	16 µM	49.5 ± 1.0 a	90.0 ± 1.9 a	0.12 ± 0.00 a
	Significance	***	***	***

*** Significant at *p* ≤ 0.001. Different letters within each column indicate significant mean differences within each genotype according to Duncan’s multiple range tests (*p* ≤ 0.05). All data are expressed as mean ± SE, *n* = 3.

**Table 4:** Phenolic profiles and total phenolic composition of four microgreen genotypes grown in a growth chamber on a capillary mat substrate under three Se concentrations applied in the nutrient solution.

Polyphenol (µg g^−1^ dw)	Coriander				Green basil				Purple basil				Tatsoi			
	0 µM Se	8 µM Se	16 µM Se	Sig.	0 µM Se	8 µM Se	16 µM Se	Sig.	0 µM Se	8 µM Se	16 µM Se	Sig.	0 µM Se	8 µM Se	16 µM Se	Sig.
cichoric acid	nd	nd	nd	na	841 ± 63.8 b	2408 ± 26.6 a	921 ± 30.0 b	***	813 ± 58.2 a	533 ± 24.3 b	481 ± 16.8 b	**	nd	nd	nd	na
chlorogenic acid	4504 ± 409	4526 ± 247	4519 ± 315	ns	208 ± 15.2 b	272 ± 14.8 a	171 ± 2.61 b	**	7.12 ± 0.18 a	6.78 ± 0.20 a	2.83 ± 0.29 b	***	12.8 ± 1.36 b	19.4 ± 1.20 a	12.4 ± 1.43 b	*
caffeic acid	0.67 ± 0.04 b	0.99 ± 0.05 a	0.86 ± 0.04 a	**	11.2 ± 0.80 b	17.1 ± 1.01 a	8.88 ± 0.24 b	***	10.89 ± 0.86 a	6.33 ± 0.43 b	5.82 ± 0.16 b	***	0.70 ± 0.06 c	0.87 ± 0.02 b	1.35 ± 0.05 a	***
kaempferol-3-*O*-sophoroside-7-*O*-glucoside	nd	nd	nd	na	nd	nd	nd	na	nd	nd	nd	na	0.66 ± 0.01 c	2.47 ± 0.17 b	7.67 ± 0.46 a	***
quercetin-sophoroside	1.46 ± 0.02 a	1.23 ± 0.04 b	0.85 ± 0.02 c	***	0.65 ± 0.03 b	1.49 ± 0.02 a	0.42 ± 0.03 c	***	0.15 ± 0.00 a	0.11 ± 0.01 b	0.08 ± 0.00 c	***	12.4 ± 0.65 b	26.0 ± 3.20 a	23.9 ± 1.47 a	**
caffeic acid hexoside isomers	39.9 ± 0.68 a	12.0 ± 0.19 c	17.7 ± 1.09 b	***	6.26 ± 0.71 c	17.6 ± 0.73 a	9.45 ± 0.73 b	***	13.0 ± 0.62 a	7.34 ± 0.71 b	12.0 ± 0.67 a	**	212 ± 8.92 b	194 ± 20.1 b	367 ± 5.93 a	***
2-xylosylvitexin	nd	nd	nd	na	nd	nd	nd	na	5.04 ± 0.56	4.12 ± 0.27	4.04 ± 0.14	ns	0.94 ± 0.08 b	0.85 ± 0.04 b	1.23 ± 0.06 a	*
kaempferol-3-O(caffeoyl)sophoroside-7-*O*-glucoside	nd	nd	nd	na	0.09 ± 0.00 b	0.15 ± 0.00 a	0.07 ± 0.01 c	***	nd	nd	nd	na	94.6 ± 6.25 b	93.2 ± 7.66 b	190 ± 1.88 a	***
isorhamnetin-3-gentiobioside	nd	nd	nd	na	nd	nd	nd	na	nd	nd	nd	na	18.9 ± 0.96 b	18.7 ± 0.50 b	39.2 ± 1.99 a	***
kaempferol-3-*O*-(coumaroyl)soph-7-*O*-glucoside	0.81 ± 0.04	0.11 ± 0.01	0.04 ± 0.00	***	0.01 ± 0.00 c	0.02 ± 0.00 a	0.02 ± 0.00 b	***	18.8 ± 0.62 a	8.48 ± 0.25 b	7.42 ± 0.63 b	***	7.09 ± 0.46 b	7.62 ± 0.38 b	15.3 ± 1.03 a	***
luteolin-7-*O*-glucoside	1.48 ± 0.01 b	1.47 ± 0.01 b	1.60 ± 0.05 a	*	14.0 ± 1.00 b	31.3 ± 1.19 a	30.2 ± 1.09 a	***	39.6 ± 0.39 a	24.4 ± 0.67 b	22.7 ± 0.94 b	***	2.18 ± 0.26 b	2.69 ± 0.14 b	4.92 ± 0.07 a	***
apigenin-malonil-glucoside	0.04 ± 0.00 b	0.05 ± 0.00 b	0.07 ± 0.00 a	***	0.34 ± 0.01 c	0.76 ± 0.03 a	0.47 ± 0.02 b	***	1.10 ± 0.04	0.99 ± 0.02	1.04 ± 0.03	ns	0.05 ± 0.00 a	0.02 ± 0.00 c	0.04 ± 0.00 b	***
kaempferol-3-O(feruoyll)sophoroside-7-*O*-glucoside	4.65 ± 0.29 a	0.31 ± 0.01 b	0.06 ± 0.00 b	***	0.03 ± 0.00 a	0.02 ± 0.00 b	0.03 ± 0.00 a	***	0.59 ± 0.04 a	0.26 ± 0.02 b	0.27 ± 0.01 b	***	42.1 ± 2.54 b	58.6 ± 3.29 b	108 ± 7.91 a	***
coumaroyl quinic acid	3.21 ± 0.06 a	0.60 ± 0.06 b	0.10 ± 0.01 c	***	0.02 ± 0.00 c	0.10 ± 0.01 b	0.14 ± 0.00 a	***	0.05 ± 0.00 a	0.03 ± 0.00 b	0.01 ± 0.00 c	***	12.3 ± 0.23 b	15.6 ± 1.21 b	38.7 ± 1.68 a	***
rutin	3487 ± 48.9 c	3857 ± 35.7 b	4653 ± 86.8 a	***	31.2 ± 0.19 a	13.3 ± 0.57 b	14.1 ± 0.63 b	***	3.42 ± 0.06	3.11 ± 0.20	3.23 ± 0.05	ns	3.16 ± 0.29 b	1.96 ± 0.05 b	2.37 ± 0.02 a	**
apigenin-7-*O*-rutinoside	0.30 ± 0.02 a	0.12 ± 0.05 b	0.01 ± 0.00 c	***	nd	nd	nd	na	nd	nd	nd	na	4.55 ± 0.19 c	6.93 ± 0.31 b	12.8 ± 0.35 a	***
quercetin-3-*O*-glucoside	88.9 ± 5.38 b	118 ± 2.32 a	79.8 ± 5.60 b	**	4.08 ± 0.03 b	4.98 ± 0.16 a	1.61 ± 0.03 c	***	39.2 ± 1.02 a	16.0 ± 0.89 b	6.83 ± 0.60 c	***	0.54 ± 0.00 c	1.26 ± 0.01 b	3.30 ± 0.03 a	***
feruloyl quinic acid	613 ± 11.7 c	734 ± 34.2 b	1123 ± 43.3 a	***	2.78 ± 0.04 c	39.6 ± 0.80 a	9.67 ± 0.17 b	***	13.4 ± 1.02 a	5.99 ± 0.17 b	5.55 ± 0.15 b	***	4.65 ± 0.20 b	8.48 ± 0.29 a	4.99 ± 0.40 b	***
rosmarinic acid	nd	nd	nd	na	10969 ± 765 b	13025 ± 128 a	11246 ± 71 b	*	11532 ± 428 a	9236 ± 246 b	9081 ± 176 b	**	nd	nd	nd	na
cirsiliol	nd	nd	nd	na	44.9 ± 1.44 c	116 ± 2.17 a	67.3 ± 0.85 b	***	31.9 ± 1.53 a	18.9 ± 0.49 b	20.1 ± 0.64 b	***	nd	nd	nd	na
ferulic acid	32.7 ± 0.97 b	36.4 ± 0.94 b	55.8 ± 2.19 a	***	25.5 ± 0.54 b	50.3 ± 2.34 a	21.6 ± 0.76 b	***	36.0 ± 1.29 a	24.9 ± 0.66 b	22.0 ± 0.24 b	***	0.47 ± 0.01 c	0.84 ± 0.06 b	1.02 ± 0.01 a	***
luteolin-3-*O*-rutinoside	248 ± 5.90 c	532 ± 15.5 a	374 ± 14.6 b	***	4.56 ± 0.03 a	3.99 ± 0.13 b	2.52 ± 0.06 c	***	15.1 ± 0.65 a	10.4 ± 0.32 b	10.2 ± 0.59 b	***	nd	nd	nd	na
kaempferol-7-*O*-glucoside	3.06 ± 0.13 a	2.23 ± 0.02 c	2.65 ± 0.05 b	***	2.50 ± 0.13 a	1.79 ± 0.03 b	1.74 ± 0.09 b	**	13.4 ± 1.15 a	9.52 ± 0.48 b	9.23 ± 0.25 b	*	3.30 ± 0.09 c	4.02 ± 0.28 b	8.38 ± 0.15 a	***
dicaffeoylquinic acid	10.7 ± 1.28 b	15.6 ± 0.42 a	9.60 ± 0.35 b	**	1.16 ± 0.04 b	3.09 ± 0.10 a	2.83 ± 0.13 a	***	0.95 ± 0.03 a	0.67 ± 0.04 b	0.42 ± 0.04 c	***	nd	nd	nd	na
kaempferol-3-*O*-rutinoside	270 ± 9.55 c	313 ± 4.63 b	408 ± 9.77 a	***	4.48 ± 0.03 b	3.23 ± 0.14 c	5.13 ± 0.18 a	***	15.1 ± 0.66 a	10.3 ± 0.34 b	10.1 ± 0.60 b	***	0.10 ± 0.01 c	0.22 ± 0.01 b	0.28 ± 0.02 a	***
quercetin-rhamnoside	0.19 ± 0.02 a	0.16 ± 0.02 ab	0.11 ± 0.00 b	*	52.1 ± 2.81	96.7 ± 3.79	148 ± 112	ns	102 ± 9.49 a	68.9 ± 1.16 b	65.0 ± 3.54 b	**	0.15 ± 0.01 c	0.25 ± 0.02 b	0.33 ± 0.01 a	***
luteolin-malonil-hexose	0.96 ± 0.08	1.04 ± 0.11	1.05 ± 0.10	ns	nd	nd	nd	na	nd	nd	nd	na	0.83 ± 0.10 b	0.91 ± 0.05 b	1.15 ± 0.03 a	*
apigenin-7-rhamnoside-4-rutinoside	nd	nd	nd	na	nd	nd	nd	na	nd	nd	nd	na	7.12 ± 0.36 c	16.1 ± 0.15 a	13.2 ± 0.17 b	***
apigenin-7-*O*-glucoside	0.05 ± 0.01	0.06 ± 0.01	0.05 ± 0.00	ns	31.6 ± 0.27 b	46.9 ± 2.36 a	21.2 ± 0.18 c	***	5.71 ± 0.43 a	3.20 ± 0.12 b	2.96 ± 0.11 b	***	0.10 ± 0.01	0.10 ± 0.01	0.08 ± 0.01	ns
Total polyphenols	9311 ± 385 b	10154 ± 311 ab	11246 ± 402 a	*	12255 ± 818 b	16154 ± 113 a	12683 ± 199 b	**	12717 ± 489 a	9999 ± 273 b	9774 ± 201 b	**	441 ± 21 b	481 ± 26 b	858 ± 8 a	***

ns, *, **, *** Non-significant or significant at *p* ≤ 0.05, 0.01, and 0.001, respectively. Different letters within each column indicate significant mean differences within each genotype according to Duncan’s multiple range tests (*p* ≤ 0.05). All data are expressed as mean ± SE, *n* = 3. nd not detected, na not available.
